# Risk Assessment of Kumasi Metropolis Population in Ghana through Consumption of Fish Contaminated with Formaldehyde

**DOI:** 10.1155/2018/4785031

**Published:** 2018-11-01

**Authors:** Noah Kyame Asare-Donkor, Raymond Akanwi Adaagoam, Ray Bright Voegborlo, Anthony Apeke Adimado

**Affiliations:** Department of Chemistry, Kwame Nkrumah University of Science and Technology, Kumasi, Ghana

## Abstract

This study evaluates the exposure of the Ghanaian population of the Kumasi Metropolis of Ghana to formaldehyde through the consumption of fish using 3-Methyl-2-Benzothiazoline Hydrazone method, with trichloroacetic acid as an extracting agent. A total of sixty (60) fish species comprising both local and imported fish were bought from cold stores and fish ponds were analysed. Formaldehyde was found in all the species analysed with concentration ranging from 0.174 to 3.710 *μ*gg^−1^. However, the levels were still lower than 5 mg/kg, which is the maximum limit established by the Malaysian Food Act and Regulation for formaldehyde in fish. The estimated daily intake values for formaldehyde in the fish species analysed ranged between 4.233 × 10^−4^ and 3.661 × 10^−3^ mg/kg BW/day and this was less than the acceptable daily intake of 0.15 and 0.2 mg/kg BW/day suggested by World Health Organization and the United States Environmental Protection Agency for formaldehyde intake, respectively. The results for the hazard quotient calculated for all the species were less than one suggesting that the amount of formaldehyde in the fish is not likely to pose any potential adverse health effects to consumers. Thus, wet fish from Kumasi may be considered safe for consumption because of low formaldehyde content.

## 1. Introduction

Fish is a prime source of protein, which is an important part of a healthy human diet worldwide [1, 2]. Fish consumption can lead to a reduction in the level of cholesterol, the occurrence of stroke, and protection against heart diseases [3, 4] as well as enhancing cognitive development of young children [5]. The free amino acids, fat, and water in fish make it prone to spoilage by biochemical reactions and microorganisms during postmortem process [6, 7]. During deterioration of some fish species, high concentrations of formaldehyde may form but do not accrue in the tissue because of subsequent conversion to other compounds. However, it may accumulate in some species such as cod, Pacific hake, Pollack, and haddock during the frozen storage as a result of the difference in the levels of trimethylamine oxide and its degradation [8]. Trimethylamine oxide demethylase (TMAOase) is an enzyme very active at low temperatures such as -20°C and is accountable for the demethylation of trimethylamine oxide to dimethylamine and FA [9]. Therefore, trimethylamine oxide breakdown helps in fish lipid oxidation, formaldehyde cross-linking leading to the toughness of protein, and the formation of dimethylamine give off the fishy odour [10]. It has also been observed that when fish undergoes postmortem changes, the TMAO gets broken down into trimethylamine, dimethylamine, and formaldehyde as the main products. It has been reported by Tunhun et al. [11] that TMAO is much more available in marine fish than in freshwater fish.

The World Health Organization (WHO) has set up a maximum daily reference dose (RfD) of 0.15 mg/kg body weight per day for formaldehyde [12] and the United States Environmental Protection Agency (USEPA) also set RfD of 0.2 mg/kg body weight per day for formaldehyde [4, 13]. Also, the Malaysian Food and Regulation have set an FA threshold limit in fish and its products to be 5 mg/kg body weight per day [4]. Intake of higher levels than the RfD may lead to a potential adverse effect on human health [14].

In Ghana, the fishery sector plays a significant role in terms of job-creating opportunities and animal protein supply [15, 16]. Fish accounts for an average of 60% of the total animal protein in Ghanaian dietary intake [15, 17–19]. There have been media reports and consumer complaints of late in Ghana of the use of formaldehyde for preserving fish regardless of the public health consequences [16, 20]. The addition of FA to fish composition can generate a chemical reaction, which could form toxic compounds or substance with hazardous effects to the consumer's health [15].

Formaldehyde, though important in many aspects of human life and endeavours, can have negative effects on human health. Formaldehyde can affect human health, particularly on the immune, blood, and central nervous systems [21]. Formaldehyde can enter the human body through ingestion, inhalation, and dermal contact. It is an important metabolic mediator in mammalian cells, which are formed during amino acids metabolism in the body [22]. The presence of formaldehyde in human system can cause minor to serious problems such as irritation of the respiratory tract, pain, vomiting, cancer, abnormalities in chromosomes, blindness, asthma, damage to the kidney, uncontrolled cell development or cancer in the stomach and gastrointestinal tract, coma, and possible death with large formaldehyde dosage [2, 4, 8, 23].

Consumption of formaldehyde-contaminated foods can lead to oxidative stress on the reproductive system due to disparity among extreme production of reactive oxygen species and inadequate antioxidant defence [24]. The enzyme formaldehyde dehydrogenase, which is found in all human tissues like red blood cells and liver, quickly transform formaldehyde to formate after which the carbon atom can be further oxidised to CO_2_ or integrated into biological macromolecules through tetrahydrofolate-dependent one-carbon biosynthetic pathway [8, 22, 25]. Commercial fish may be contaminated with formaldehyde used as a preservative in order to keep the freshness of wet fish and seafood because they are very perishable and can only be kept fresh in ice for a few days depending on the species [7]. In addition there have been media reports of late in Ghana of alleged use of formaldehyde to aid preservation in the processing of salted fish which is a delicacy in Ghana, by the fishermen and fish mongers. Apart from a qualitative study which sought to establish the presence of formaldehyde in imported and local fresh fish in the Tamale Metropolis of Ghana [16], research regarding the presence and assessment of exposure and risk of formaldehyde through fish consumption in Ghana is nonexistent. Since formaldehyde is a known carcinogen as well as having other negative human health related effects coupled with the fact that it had been reported to be a major contaminant in seafood and fish, this work seeks to investigate formaldehyde content in fish and assess the health risk on consumption.

## 2. Materials and Methods

### 2.1. Sampling and Sample Preparation

The study was conducted on fresh fish from various cold stores with freezer storage temperature range from -10°C to -20°C and fishponds in Kumasi Metropolis of Ghana. The fish species were obtained from four retail cold stores (coded: A, B, C, D), three wholesale cold stores (coded: E, F, G), and two fishponds (coded: H, I) in Kumasi Metropolis of Ghana depending on the species available at the time of sampling. A total of sixty (60) fish comprising nineteen (19) different species were obtained from the various sampling points on different days and were transported on an ice bath to Kwame Nkrumah University of Science and Technology (KNUST) Fisheries Department for scientific identification and later brought to the (KNUST) Chemistry Department Laboratory for analysis. Each fish sample was unfrozen and then cut into small pieces and put in zipped clean plastic bags and kept in a deep freezer for three days pending extraction and analysis.

### 2.2. Formaldehyde Determination

The spectrophotometric determination of the formaldehyde content in the fish samples were determined using a method described by Sibirnyi et al. [26, 27] with some modification. Briefly about 30 g of each fish sample was homogenized for 10 minutes with a homogenizer (IKA T18 basic, ULTRA-TURRAX). 60 ml of 6% (w/w) trichloroacetic acid was added to extract the formaldehyde from the fresh fish and allowed to stand for 30 minutes at ambient temperature. The extracted solution was then filtered with a Whatman No. 1 filter paper. 5 ml of the filtered fish extract solution was taken and 5 ml of 0.05% (w/v) MBTH was added followed by 5 ml of 100 ml solution of 1.0 g ferric chloride and 1.6 g sulphamic acid was then added and diluted to the mark. The solution was allowed to stand at room temperature for 15 min and the absorbance was measured at 628 nm using UVmini-1240 UV-Vis Spectrophotometer (SHIMADZU). Blank solution was prepared by replacing the fish extract with distilled water and treating it in the same manner as the sample. During the calibration process, a standard formaldehyde solution in the range of 0 to 2.5 mg/l was prepared from a 10 mg/l stock solution. The amount of formaldehyde in the fish sample (*μ*g g^–1^) was calculated from standard formaldehyde curve.

### 2.3. Recovery Test

For quality control, an analytical spike recovery was done by adding standard formaldehyde solutions prepared from 37% formaldehyde to check for the reliability of the methods used. That is, a known amount of the analyte was added to the fish samples before extraction and also to the extracts and was analysed using UVmini-1240 UV-Vis Spectrophotometer. The percentage recovery was calculated to assess the accuracy of the method. To assess the precision, all fish extract analyses were performed in duplicate and the absorbance for each taken three times. The mean absorbance was then used to calculate the formaldehyde concentration from the calibration curve to ensure the quality of the results. Reagent blank was also prepared by replacing the fish extract with distilled water and treating it in a similar manner as the samples in order to minimise errors and to ensure the quality of the results.

### 2.4. Deterministic Risk Assessment

FA can stimulate mutations of gene and aberration of the chromosome in the cells of mammals. It can cause protein-DNA cross-linkage, which is a sensitive estimation of the modification of DNA [4]. Monitoring and assessment of the hazard associated with the health of human from consumption of FA contaminated fish will require information on the quantities of fish consumed per person per day [4]. The variety of fish consumed may differ significantly from one person to another and also from country to country. The quantity of FA intake daily as a result of fish consumption depends on the type of fish and the amount consumed [4]. In Ghana, the estimated average fish consumption per capita is about 27 kg per annum [28]. The values of EDI were determined with the assumption that a person weighing 75 kg is likely to consume 74 g/day of fish [4, 29].

### 2.5. Estimated Daily Intake (EDI) Calculation

The estimated daily intake (EDI) of FA in fish was done by calculating the amount of FA in fish in order to determine the hazard quotient (HQ). The EDI calculation is given in [30]

(1) $$\begin{array}{c} EDI=\frac{Cm\left(mg/kg\right)\times IR\left(kg/day\right)}{BW\left(kg\right)} \end{array}$$

where EDI is the estimated daily intake (mg kg^–1^ day^–1^), Cm is the concentration of contaminant (formaldehyde in fish tissue wet weight)/mg/kg or *μ*g/g, IR is the ingestion or consumption rate, and BW is the body weight (75 kg).

### 2.6. The Hazard Quotient (HQ)

The HQ calculation is given in [31]

(2) $$\begin{array}{c} HQ=\frac{EDI}{RfD} \end{array}$$

where HQ is the hazard quotient and RfD is the reference dose (mg kg^–1^ day^–1^).

HQ values of < 1 signify unlikely adverse health effects, while HQ values >1 indicate a likely adverse health effect.

## 3. Statistical Analysis

The mean and standard deviation of the formaldehyde concentration was determined using the statistical computer software. Statistical Package for Social Science version 16 was used in this study. The results were subjected to one-way ANOVA to compare the mean amount of FA in the fish followed by post hoc using Tukey HSD tests at* p* < 0.05.

## 4. Results and Discussion

### 4.1. Amount of Formaldehyde in Fish

The results presented in Table 1 indicate that all the fish species contain certain amount of formaldehyde which differed among the fish species. Comparisons of the mean amount of FA among all the fish species analysed showed a significant difference (*p* < 0.05) for FA among the fish species analysed. The concentration of formaldehyde in the imported fish species ranged between 0.701 and 3.710 *μ*g g^–1^ (Table 1). These results are comparable with some of the reported for finfish and shellfish samples from different markets in Dhaka in the range of 0.33 to 16 mgkg^−1^ [1, 7, 31–33]; Jannan et al., 2015. These values are however lower than the results obtained by Bhowmik et al. [34] in freshwater and marine finfish samples and shrimp in Bangladesh who obtained FA content in the range of 5.1 ± 0.71 to 39.68 ± 7.87 mgkg^−1^ in all marketed fish. The disparity of FA for all types of fish samples analysed could be explained on the bases that different species have a different amount of trimethylamine oxide (TMAO) even if intentional addition of FA to prolong the shelf life and maximised profit was not considered. The natural occurrence of formaldehyde in fish, which might form as a result of the enzymatic breakdown of TMAO to FA and dimethylamine (DMA), also elevates the activity of other microorganisms in the fish [4, 8, 35]. The type of fish species, source, duration, and temperature of storage mostly determined the quantity of formaldehyde produced, which causes toughening of muscle and loss of water in fish, reducing the fish quality [4, 35, 36]. Saltwater species, such as Pacific hake, cod, Pollack, and haddock, contains a high amount of TMAO as a natural constituent, which was used for cell osmoregulation [37]. TMAO could undergo enzymatic breakdown to produce DMA and FA with the aid of the enzyme TMAO-ase. Hence, these species might contain high levels of FA because of TMAO breakdown and not FA adulteration. These make it hard to differentiate naturally occurring formaldehyde from contamination in the Gadidae family species [37, 38]. Some fish species (e.g., herring fish) also contain dark muscle tissue, which was one of the factors that could also lead to the formation of FA in some fish species. This muscle was situated along the side of its body next to the skin and it contains a high content of fat, oxygen, and red blood cells. It is believed that dark muscle of marine fish contained a large number of nitrogenous substances than the white muscle [39]. The mixture of oxygen and fat could make fish extra prone to lipid oxidation or rancidity. Several nitrogenous substances in dark muscle tissue contain a larger quantity of TMAO, dipeptides, free amino acids, and imidazole, and the breakdown of TMAO could lead to higher levels of FA. Therefore, the larger the dark muscle was, the more liable the fish was towards spoilage [39]. Notwithstanding, the levels found in the fish samples were far less than the limit 5 *μ*g g^–1^ established by the Malaysian Food Act and Regulation for formaldehyde in fish and its products [4]. Also, formaldehyde content in local tilapia (Table 2) ranged between 1.118 and 2.430 *μ*g g^–1^ and the fresh fish from ponds (tilapia and catfish), which might be presumed to contain natural formaldehyde ranged between 0.428 and 1.580 *μ*g g^–1^ (Table 3), which was rather lower than that of the local tilapia from central market and the imported fish species. These variations in the formaldehyde content in the same fish species from the different sources and among the different species of fish could be attributed to fish habitat, storage time, storage temperature, compositional differences, and differences in response to reaction between fish protein and formaldehyde [40].

Also, the results showed no significant difference between the FA content in imported and the local fish (tilapia and catfish) sampled from the cold stores and the fish ponds in Kumasi from the results of the one-way ANOVA analysis (Table 4).

### 4.2. Risk Assessment of Formaldehyde Content of Fish Species Analysed

The assessment of estimated daily intake and the hazard quotient for formaldehyde in the fish sample analysed shown in Table 5 indicated that the risks calculated as HQ were the ratio of the estimated daily intake (EDI) of formaldehyde in fish to the WHO daily RfD of 0.15 mg/kg BW/day [41].

The RfDs signify an approximation of human daily consumption of fish above which consumers might be constantly exposed to significant health threat. The EDI values for the formaldehyde in the different types of fish species analysed ranged between 4.233 × 10^–4^ and 3.661 × 10^–3^ mg/kg BW/day, which was all far less than 0.15 and 0.2 mg/kg BW/day limit set by WHO and USEPA, respectively. From the results (Tables 5, 6, and 7) all HQ values for all the fish species analysed were less than one (HQ < 1), which signifies that the level of formaldehyde in fish samples was not likely to have potential adverse health effects to the consumer.

## 5. Conclusion

The investigation indicated that there was FA in all the fish species analysed and this ranged between 0.174 and 3.710 *μ*g g^–1^ which was far lower than the maximum limit of 5 mg/kg set by the Malaysian Food Act and Regulation for formaldehyde in fish and its products. The EDI values for FA in the fish species ranged from 4.233 × 10^–4^ to 3.661 × 10^–3^ mg/kg BW/day, which was also lower than the maximum daily RfD of 0.15 and 0.2 mg/kg BW/day for formaldehyde established by the WHO and the United States EPA, respectively, and hence not of regulatory concern. The risk assessment from the study indicated that the HQ computed for all the species were far below 1. This signifies that the amount of FA in the fish samples is not likely to cause any potential adverse health effects to the consumer. Therefore, fresh fish in Kumasi Metropolis of Ghana does not contain high levels of FA per the study results. Thus, fresh fish species from Kumasi Metropolis of Ghana, during the study period, might not have been treated with FA as a preservative.

## Figures and Tables

**Table 1:** Mean FA concentration and standard deviation (SD) in fish from retail and wholesale cold stores.

Fish Name	Scientific Name of Fish	Mean ± SD (*μ*g g^–1^)						
		A	B	C	D	E	F	G
Bluefish	*Pomatomus saltatrix*	–	–	–	–	1.10 ± 0.04	1.67 ± 0.04	1.73 ± 0.05
Butterfish	*Stromateus fiatola*	1.65 ± 0.00	1.47 ± 0.73	–	–	–	–	–
Cassava Croaker	*Pseudotolithus senegalensis*	1.24 ± 0.02	–	–	–	–	–	–
Dark Redfish	*Brachydeuterus auritus*	–	–	0.92 ± 0.15	–	–	–	–
European Barracuda	*Sphyraena sphyraena*	1.26 ± 0.02	2.39 ± 0.22	–	–	–	–	–
Herrings	*Clupea harengus*	2.96 ± 0.00	–	–	–	2.20 ± 0.01	2.00 ± 0.16	–
Horse Mackerel	*Trachurus trachurus*	1.71 ± 0.11	0.80 ± 0.02	–	–	2.79 ± 0.11	1.62 ± 0.10	2.51 ± 0.08
Pacific Hake (kako)	*Merluccius productus*	–	–	3.71 ± 0.43	–	1.26 ± 0.05	0.96 ± 0.14	0.98 ± 0.10
Redfish	*Pagellus affinis*	1.25 ± 0.16	1.98 ± 0.18	–	1.08 ± 0.02	2.98 ± 0.02	2.48 ± 0.64	2.86 ± 0.10
Salmon	*Oncorhynchus *spp.	1.99 ± 0.05	1.76 ± 0.20	1.51 ± 0.08	3.34 ± 0.10	1.69 ± 0.14	1.77 ± 0.04	1.44 ± 0.23
Salmonete	*Mullus barbatus*	–	–	–	–	1.97 ± 0.33	3.61 ± 0.02	2.96 ± 0.02
Sea bream	*Sparus *spp.	1.20 ± 0.13	–	–	–	1.21 ± 0.12	1.64 ± 0.02	–
Sole Fish	*Cynoglossus macrolepidotus*	–	1.45 ± 0.04	–	–	–	–	–
African moonfish	*Selene dorsalis*	–	1.42 ± 0.14	–	–	1.21 ± 0.12	–	2.07 ± 0.16
Threadfin fish (sukwei)	*Galeoides decadactylus*	–	–	–	–	2.85 ± 0.12	–	2.21 ± 0.14
Warwas Grouper	*Epinephelus nigritus*	–	–	–	–	2.73 ± 0.30	–	–
Catfish	*Clarias gariepinus*	–	–	–	–	–	0.70 ± 0.05	–
Cassava fish	*Pseudotolithus brachygnathus*	–	–	–	–	–	–	2.97 ± 0.02
Glasseye Snapper	*Pricanthus arenatus*	–	–	–	–	–	–	3.03 ± 0.54

**Table 2:** Mean FA concentration and standard deviation (SD) of local fish *Oreochromis niloticus* (tilapia) sampled from retail and wholesale cold stores.

Sampling point	Mean ± SD (*μ*g g^–1^)
LTA	1.118 ± 0.023
LTB	2.430 ± 0.013
LTC	1.220 ± 0.011
LTD	2.255 ± 0.001
LTE	2.418 ± 0.031
LTF	1.515 ± 0.076

LTA= local tilapia at point *A*, LTB = local tilapia at point *B*, LTC = local tilapia at point *C*, LTD = local tilapia at point *D*, LTE = local tilapia at point *E*, and LTF = local tilapia at point *F*.

**Table 3:** Mean FA concentration and standard deviation (SD) in fish from ponds.

Fishponds	Mean ± SD (*μ*g g^–1^)
Pond H. *Clarias gariepinus* (Catfish)	0.648 ± 0.009
Pond H. *Oreochromis niloticus* (Tilapia)	1.580 ± 0.037
Pond I. *Oreochromis niloticus* (Tilapia)	0.615 ± 0.008
Pond I. *Clarias gariepinus* (Catfish)	0.428 ± 0.028

**Table 4:** One-way ANOVA results for the fish species from the various cold stores/fish ponds.

Cold store/Fish ponds		Sum of squares	df	Mean square	F	Sig.
A	Between groups	5.009	7	0.716	101.496	0.000
	Within groups	0.056	8	0.007		
	Total	5.065	15			
B	Between groups	3.026	6	0.504	5.282	0.023
	Within groups	0.668	7	0.095		
	Total	3.695	13			
C	Between groups	8.626	2	4.313	60.101	0.004
	Within groups	0.215	3	0.072		
	Total	8.841	5			
D	Between groups	2.596	5	0.371	92.605	0.000
	Within groups	0.032	8	0.004		
	Total	2.628	13			
E	Between groups	11.124	10	1.112	44.326	0.000
	Within groups	0.276	11	0.025		
	Total	11.400	21			
F	Between groups	11.493	8	1.437	27.668	0.000
	Within groups	0.467	9	0.052		
	Total	11.961	17			
G	Between groups	9.294	9	1.033	24.377	0.000
	Within groups	0.424	10	0.042		
	Total	9.718	19			
H	Between groups	3.73	5	0.746	594.246	0.000
	Within groups	0.008	6	0.001		
	Total	3.737	11			
I	Between groups	1.603	3	0.534	925.447	0.000
	Within groups	0.002	4	0.001		
	Total	1.606	7			

**Table 5:** The EDI results for carcinogenic risk evaluation expressed in mg/kg body weight/day.

	A		B		C		D		E		F		G	
Fish Name	EDI	HQ	EDI	HQ	EDI	HQ	EDI	HQ	EDI	HQ	EDI	HQ	EDI	HQ
Bluefish	1.632	10.880	1.445	9.633	-	-	-	-	1.084	7.229	1.645	1.0.97	1.702	11.35
Butterfish														
Cassava Croaker	1.224	8.160	-	-	-	-	-	-	-	-	-	-	-	-
Cassava fish	-	-	-	-	-	-	-	-	-	-	-	-	2.931	19.54
Catfish	-	-	-	-	-	-	-	-	-	-	0.692	4.611	-	-
Dark Redfish	-	-	-	-	0.910	6.065	-	-	-	-	-	-	-	-
European Barracuda	1.247	8.314	2.359	1.573	-	-	-	-	-	-	-	-	-	-
Glasseye Snapper	-	-	-	-	-	-	-	-	-	-	-	-	2.988	19.92
Herrings	2.920	19.46	-	-	-	-	-	-	2.172	14.48	1.974	13.16	-	-
Horse Mackerel	1.688	11.25	0.785	5.233					2.752	18.35	1.599	10.66	2.478	16.52
Pacific Hake (Kako)	-	-	-	-	3.661	24.40	1.064	7.091	1.245	83.01	0.949	6328	9.699	6.466
Redfish	1.238	8.252	1.953	13.02	-	-	-	-	2.935	19.57	2.443	16.29	-	-
Salmon	1.964	13.10	1.732	11.55	1.495	9.965	3.295	21.97	1.667	11.12	1.749	11.66	1.417	9.446
Salmonete	-	-	-	-	-	-	-	-	1.945	12.96	3.561	23.74	2.923	19.49
Sea bream	-	-	-	-	-	-	-	-	1.195	7.966	1.623	10.82	-	-
Sole Fish	-	-	1.428	9.518	-	-	-	-	-	-	-	-	-	-
African moonfish	1.187	7.913	1.402	9.347	-	-	-	-	1.197	7.979	-	-	2.046	13.64
Threadfin fish (Sukwei)	-	-	-	-	-	-	-	-	2.810	1.873	-	-	2.176	14.50
Warwas Grouper	-	-	-	-					2.694	1.796	-	-	-	-

The EDI and HQ values are to the power 10^–3^.

**Table 6:** The EDI results for carcinogenic risk evaluation expressed in mg/kg body weight/day for tilapia in cold stores.

**SAMPLING POINT**	**WHO RfD**	**FA**	**EDI**	**HQ**
	**(mg/kg BW/day)**	**(** ***μ*** **gg** ^-**1**^ **)**	**(mg/kg BW/day)**	
LTA	0.15	1.118	1.103 × 10^−3^	7.354 × 10^−3^
LTB	0.15	2.430	2.398 × 10^−3^	1.598 × 10^−2^
LTC	0.15	1.220	1.204 × 10^−3^	8.025 × 10^−3^
LTD	0.15	2.255	2.225 × 10^−3^	1.483 × 10^−2^
LTE	0.15	2.418	2.386 × 10^−3^	1.591 × 10^−2^
LTF	0.15	1.516	1.496 × 10^−3^	9.972 × 10^−3^

LTA= local tilapia at sample point **A**, LTB= local tilapia at sample point **B**, LTC= local tilapia at sample point **C**, LTD= local tilapia at sample point **D**, LTE= local tilapia at sample point **E**, and LTF= local tilapia at sample point **F**.

**Table 7:** The EDI results for carcinogenic risk evaluation expressed in mg/kg body weight/day for tilapia and catfish from fish ponds (H, I and J).

**FISH TYPE**	**WHO RfD**	**FA**	**EDI**	**HQ**
	**(mg/kg BW/day)**	**(** ***μ*** **gg** ^-**1**^ **)**	**(mg/kg BW/day)**	
Pond H. Catfish	0.15	0.648	6.394 × 10^−4^	4.262 × 10^−3^
Pond H. Tilapia	0.15	1.580	1.558 × 10^−3^	1.039 × 10^−2^
Pond I. Tilapia	0.15	0.616	6.078 × 10^−4^	4.052 × 10^−3^
Pond J. Catfish	0.15	0.428	4.233 × 10^−4^	2.822 × 10^−3^

## Data Availability

The data used to support the findings of this study are included within the article.

## Abbreviations

FA: Formaldehyde
MBTH: 3-Methyl-2-Benzothiazoline Hydrazone
AHMT: 4-Amino-3 Hydrazino-5-Mercapto-1, 2, 4-Triazole
TCA: Trichloroacetic acid
TMAO: Trimethylamine oxide
DMA: Dimethylamine
US EPA: United State Environmental Protection Agency
IARC: International Agency for Research on Cancer
EDI: Estimated daily intake
HQ: Hazard quotient
DNA: Deoxyribonucleic acid
RNA: Ribonucleic acid
RfD: Daily reference dose
*K*_oc_: Organic carbon/water partition coefficient
*K*_ow_: Octanol/water partition coefficient
BW: Body weight.
